# Caffeic acid methyl ester inhibits mast cell activation through the suppresion of MAPKs and NF-κB signaling in RBL-2H3 cells

**DOI:** 10.1016/j.heliyon.2023.e16529

**Published:** 2023-05-20

**Authors:** Jin-Young Park, Hee Jae Lee, Eun-Taek Han, Jin-Hee Han, Won Sun Park, Yong-Soo Kwon, Wanjoo Chun

**Affiliations:** a Department of Pharmacology, Kangwon National University School of Medicine, Chuncheon, 24341, South Korea; b Department of Medical Environmental Biology and Tropical Medicine, Kangwon National University School of Medicine, Chuncheon, 24341, South Korea; c Department of Physiology, Kangwon National University School of Medicine, Chuncheon, 24341, South Korea; d College of Pharmacy, Kangwon National University, Chuncheon, 24341, South Korea

**Keywords:** CAME, Phorbol-12-myristate-13-acetate, COX-2, NF-κB, RBL-2H3 cells, MAPKs

## Abstract

Anti-inflammatory effects of caffeic acid derivatives have been widely reported. However, the effect of caffeic acid methyl ester (CAME) on the anti-allergic effect in mast cells has not been elucidated. The present study was aimed to investigate the anti-allergic properties of CAME and its underlying mechanism. Rat basophilic leukemia (RBL-2H3) cells were incubated withphorbol-12-myristate-13-acetate (PMA) and a calcium ionophore, A23187 to induce mast cell activation. Anti-allergic effect of CAME was examined by measuring cytokine, histamine and β-hexosaminidase release. Western blotting was conducted to determine cyclooxygenase-2 (COX-2) expression, Mitogen-activated protein kinases (MAPKs) activation and nuclear factor-κB (NF-κB) translocation. CAME significantly suppressed PMA/A23187-induced TNF-α secretion, and β-hexosaminidase and histamine release in a concentration-dependent manner. Furthermore, CAME significantly attenuated PMA/A23187-induced COX-2 expression and nuclear translocation of NF-κB. CAME significantly suppressed PMA/A23187-induced increased phosphorylation of p38, ERK and JNK RBL-2H3 cells. The results demonstrate that CAME significantly attenuates anti-allergic action by suppressing degranulation of mast cells through the suppression of MAPKs/NF-κB signaling pathway in RBL-2H3 cells.

## 1 Introduction

Allergic disorders are a number of pathologic conditions caused by hypersensitivity of the immune system to allergen in a harmless environment [1]. Aberrant activation of mast cells plays a detrimental role in the initiation of allergic response in a variety of allergic diseases including asthma, atopic dermatitis, rhinitis, and anaphylaxis [[2], [3], [4]]. Activated mast cells release a broad spectrum of immune mediators including histamine, chemokines, cytokines, lipid compounds and vasoactive amines to facilitate host defense against foreign antigens [5]. In addition to their beneficial effects in host defense, abnormally activated mast cells initiate diverse pathophysiologic events including the contraction of airway smooth muscles, secretion of mucus, and recruitment of other inflammatory cells in various allergic conditions such as asthma, atopic dermatitis, allergic rhinitis and autoimmune diseases [3,4]. Therefore, pharmacological intervention of the abnormal activation of mast cells could be a promising strategy for the management of allergic diseases.

MAPKs play a key role in the production of inflammatory mediators including cytokines in activated mast cells and subsequent recruitment of other immune cells [6]. MAPKs include extracellular signal-regulated kinase (ERK), *c*-Jun *N*-terminal kinase (JNK), and p38 mitogen-activated protein kinase (p38) [7]. Activation of ERK has been demonstrated to regulate various mast cell responses such as proliferation, migration and differentiation in allergic conditions [8]. Activation of JNK has been shown to cause the expression of pro-inflammatory transcription factor AP1 [9]. Activation of p38 has been also reported to be involved in the production of pro-inflammatory cytokines in aberrantly activated mast cells by upregulating the expression of transcription factor, NF-κB [10]. NF-κB is the redox-sensitive transcription factor that is responsible for producing inflammatory mediators including cytokines and iNOS [11]. The aberrant expression of cytokines and iNOS was observed in activated mast cells and suppression of NF-kB activation significantly inhibited the characteristics of allergic responses [12].

Caffeic acid esters are present in many natural plants in the forms of ester derivatives including isopropenyl ester (CAIE), caffeic acid benzyl ester (CABE), caffeic acid phenethyl ester (CAPE), and caffeic acid methyl ester (CAME) [[13], [14], [15]] and have been reported to exhibit a wide range of biological activities such as anti-oxidant, anti-microbial, anti-inflammatory, cytotoxic, and anti-acetylcholinesterase properties [13,[16], [17], [18]]. CAPE has been reported to suppress cytokine-induced NF-κB signaling in macrophage cells [19] and plays an important role in the regulation of the host immune response [20]. Recently, CAPE has been reported to exert anti-allergic effects by inhibiting MAPK and NF-κB signaling in activated HMC-1 human mast cells [21]. CAME has been reported to exhibit a wide range of pharmacological actions such as anti-inflammatory and neuroprotective properties [22]. Given that CAME exerts wide range of anti-inflammatory actions and its derivative, CAPE possesses anti-allergic property, CAME might also exhibit anti-allergic action in mast cells. Therefore, the goal of the present study was to examine the anti-allergic properties of CAME and its underlying mechanism in PMA/A23187-challenged RBL-2H3 mast cells in order to provide an important therapeutic agent that could suppress various allergic conditions.

## 2 Materials and methods

### 2.1 Reagents and cell culture

Caffeic acid methyl ester (CAME) was isolated and identified from *Lonicera maackii* [23]. Phorbol 12-myristate 13-acetate (PMA) and A23187 were purchased from Sigma-Aldrich (St. Louis, MO, USA). Rat basophilic leukemia (RBL-2H3) cells were purchased from the Korea cell line bank (KCLB), KCLB cat #22256. RBL-2H3 cells were maintained in medium RPMI 1640 (RPMI 1640; Hyclon Laboratories) containing 10% heat-inactivated fetal bovine serum and 100 U/ml penicillin-streptomycin (Gibco) at 37 °C, 5% CO_2_. Cells were incubated in the presence of the indicated concentrations of CAME and then stimulated with 50 nM of PMA and 1 μM of A23187 for the indicated times.

### 2.2 TNF-α release assays

RBL-2H3 cells were pretreated with CAME (10–100 μM) for 1 h and then stimulated in the presence or absence of PMA/A23187 for 30min. TNF-α released into the medium of RBL-2H3 cultures was detected using enzyme-linked immunosorbent (ELISA) kits (R&D system, USA) according to the manufacturer's instructions.

### 2.3 β-Hexosaminidase and histamine release assay

To examine the effect of CAME on mast cell degranulation, the levels of β-hexosaminidase and histamine release were quantitatively measured. These enzymes are contained within granules in mast cells and has been used as granule markers [24]. RBL-2H3 cells were cultured in 12-well plates for 24 h. Then the medium was removed and the cells were incubated with different concentration of CAME diluted in PIPES buffer for 1 h at 37 °C. After pretreatment, cells were washed twice with PIPES buffer, then stimulated with PMA/A23187 for 30 min at 37 °C. And 20 μL of supernatant was allowed to react with 80 μL of substrate buffer (2 mM 4-*p*-nitrophenyl-*N*-acetyl-ββ-_d_-glucosaminide in 0.05 M sodium citrate buffer, pH 4.5) 30min at 37 °C. The reaction stopped by the addition of 200 μL of stop buffer (0.1 M NaHCO_3_, pH10). The absorbance was measured at 405 nm using microplate spectrophotometer (SpectraMax M5, Molecular Devices, USA). The amount of histamine was detected by *o*-phthalaldehyde (OPT) spectroflurometric procedure. To 0.5 mL of supernatant from each well, 0.1 mL of 1 M NaOH and 25 μL of OPT (1% (w/v) in methanol) were added. The supernatant was incubated for 4min at room temperature. The reaction stopped by the addition of 50 μL of 3 M HCl. The absorbance was measured at excitation and emission wavelengths of 360 nm and 450 nm, respectively, using microplate spectrophotometer (SpectraMax M5, Molecular Devices, USA).

### 2.4 Preparation of cytoplasmic and nuclear fractions

RBL-2H3 cells were treated with 10, 50, and 100 μM concentrations of CAME for 1 h prior to PMA/A23187 treatment. Cells were washed with ice-cold PBS, and harvested, and centrifuged at 15,000×*g* for 10min at 4 °C. Cytoplasmic and Nuclear extracts were prepared as described previously [25]. Briefly, cells were resuspended in 40 μL of a cold hypotonic buffer (10 mM HEPES/KOH, 2 mM MgCl_2_, 0.1 mM EDTA, 10 mM KCl, 1 mM DTT, and 0.5 mM PMSF, pH7.9). The cells were left on ice for 10min after which they were lysed gently with 2.5 μL of 10% Nonidet P (NP)-40. The lysate was centrifuged at 15,000×*g* for 3 min at 4 °C. The supernatant was collected and used as the cytoplasmic extract. The nuclear pellets were gently resuspended in 40 μL of cold saline buffer (50 mM HEPES/KOH, 50 mM KCl, 300 mM NaCl, 0.1 mM EDTA, 10% glycerol, 1 mM DTT, and 0.5 mM PMSF, pH 7.9) and left 20 min on ice. After centrifuge at 15,000×*g* for 15 min at 4 °C.

### 2.5 Western blot analysis

RBL-2H3 cells were incubated with CAME for 1 h or 2 h prior to PMA/A23187 treatment. Cells were washed with PBS and lysed in PRO-PREP lysis buffer (iNtRON Biotechnology, Seongnam, Korea). Equal amounts of protein were separated on 10% SDS-polyacrylamide gel. Proteins were transferred to Hypond PVDF membrane (Amersham Biosciences, Piscataway, NJ, USA) and blocked in 5% skim milk in TBST for 1 h at room temperature. Specific antibodies against COX-2, p38, p-p38, ERK, *p*-ERK, JNK, *p*-JNK, NFκB (p65), PARP (1:1000; Cell signaling Technology), and β-actin (1:2500; Sigma) were diluted in 5% skim milk. After thoroughly washing with TBST, horseradish peroxidase-conjugated secondary antibodies were applied. The blots were developed by the enhanced chemiluminescence detection (Amersham Biosciences).

### 2.6 Statistical analysis

All values shown in the figures were expressed as the mean ± SD obtained from at least three independent experiments. Statistical significance was analyzed by two-tailed Student's t-test. Data with values of *p* < *0.05* were considered as statistically significant. Single (*) and double (**/##) marks represent statistical significance in *p* < *0.05* and *p* < *0.01*, respectively.

## 3 Results

### 3.1 CAME inhibited PMA/A23187-induced TNF-α release

**Fig. 1:**
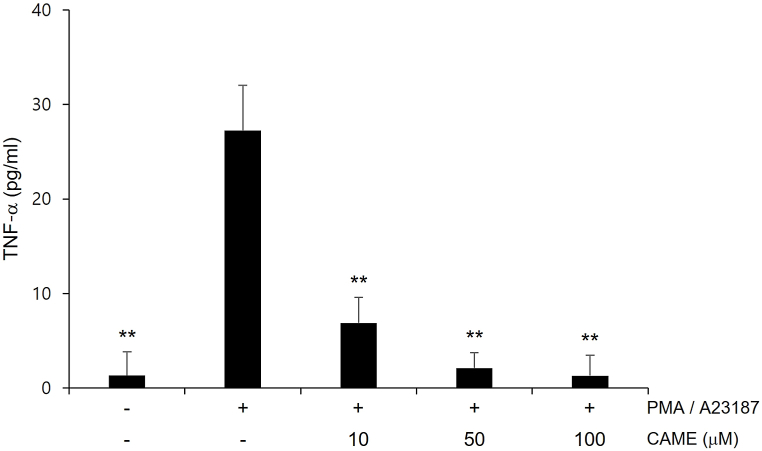
Effect of CAME on PMA/A23187-induced extracellular secretion of TNF-α in RBL-2H3 cells. RBL-2H3 cells were pretreated with indicated concentrations of CAME for 1 h, then incubated with PMA/A23187 for 30min. The concentrations of TNF-α in culture media were measured by ELISA assay as described in the methods. CAME exhibited a significant suppression of PMA/A23187-induced TNF-α secretion in a concentration-dependent manner. Data are expressed as mean ± standard deviation. *P < *0.05* and **P < *0.01* vs PMA/A23187 alone.

TNF-α, a pro-inflammatory cytokine, has been demonstrated to play a crucial role in the progression of inflammation [26,27]. The effects of CAME on the secretion of TNF-α in PMA and A23187-treated RBL-2H3 mast cells were measured. Cells were incubated with CAME for 1 h prior to PMA/A23187 treatment. PMA/A23187 treatment clearly increased the secretion of TNF-α in RBL-2H3 mast cells and CAME significantly suppressed PMA/A23187-induced TNF-α release in a concentration-dependent manner (Fig. 1). No noticeable cytotoxicity was observed with CAME treatment in the concentration ranges used in the present study (data not shown).

### 3.2 CAME inhibited PMA/A23187-induced β-hexosaminidase and histamine release

**Fig. 2:**
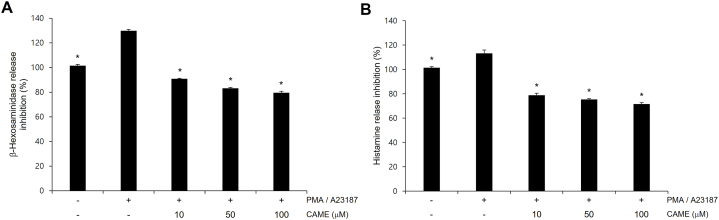
Effects of CAME on β-hexosaminidase release (A) and histamine release (B) in RBL-2H3 cells. Cells were treated with indicated of CAME for 1 h and stimulated with PMA/A23187 for 30min. CAME significantly attenuated PMA/A23187-induced β-hexosaminidase release (A) and also showed a significant suppression of PMA/A23187-induced histamine release (B) in concentration-dependent manners. Results are presented as the mean ± SD of three experiments. *P < *0.05* and **P < *0.01* vs PMA/A23187 alone.

The extracellular release of β-hexosaminidase and histamine has been reported to be a characteristic feature of cell degranulation [28]. In the present study, PMA/A23187 treatment showed the increased secretion of β-hexosaminidase and histamine. CAME treatment significantly suppressed PMA/A23187-induced secretion of β-hexosaminidase and histamine in RBL-2H3 cells in a concentration-dependent manner (Fig. 2).

### 3.3 CAME attenuated PMA/A23187-induced COX-2 expression

**Fig. 3:**
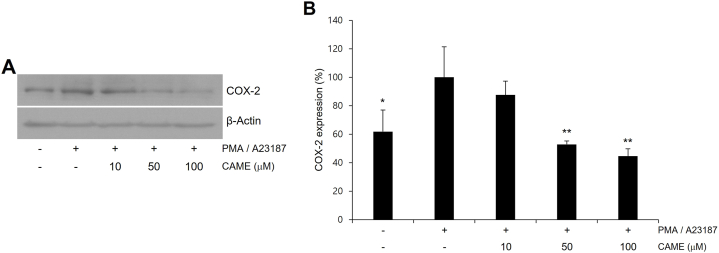
Effect of CAME on PMA/A23187-induced COX-2 expression in RBL-2H3 cells. (A) Representative immunoblots. (B) Quantitative analyses of immunoblots of COX-2. Cells were pretreated with CAME (10, 50, and 100 μM) for 2 h and then stimulated with PMA/A23187 for 30min. The expression of COX-2 was measured by Western blotting. CAME exhibited a significant attenuation of PMA/A23187-induced COX-2 expression in a concentration-dependent manner. The data presented are the means ± SD of three independent experiments. *P < *0.05*, **P < *0.01* vs. PMA/A23187 alone.

Increased expression of COX-2 has been reported to be associated with mast cell activation [20], the level of COX-2 expression was examined in PMA/A23187-challenged RBL-2H3 cells. PMA/A23187 treatment resulted in increased expression of COX-2 (Fig. 3), and CAME treatment significantly attenuated PMA/A23187-induced expression of COX-2 (Fig. 3A). Quantitative analysis of COX-2 expression showed significant suppression of COX-2 expression in a concentration-dependent manner (Fig. 3B).

### 3.4 CAME suppressed PMA/A23187-induced MAPKs phosphorylation

**Fig. 4:**
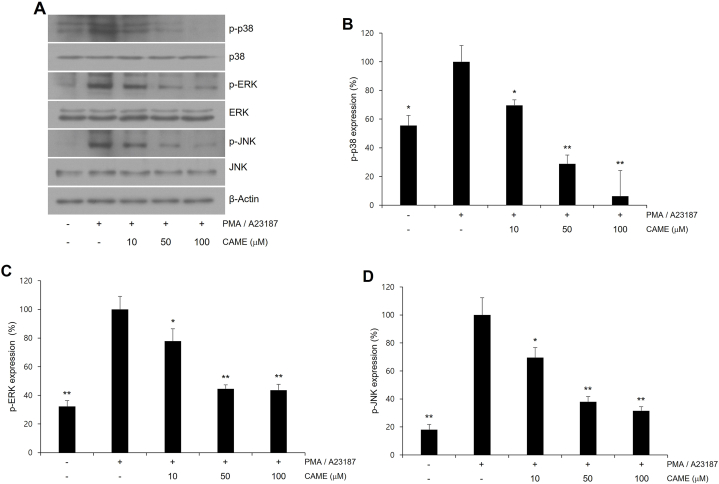
Effect of CAME on PMA/A23187-induced phosphorylation of MAPKs in RBL-2H3 cells. Cells were pretreated with CAME (10, 50, and 100 μM) for 1 h and then stimulated with PMA/A23187 for 30min. (A) Representative immunoblots of MAPKs. (B) Quantitative analyses of immunoblots of p-p38. (C) Quantitative analyses of immunoblots of *p*-ERK. (D) Quantitative analyses of immunoblots of *p*-JNK. CAME exhibited a significant suppression of PMA/A23187-induced MAPKs activation. p-p38 (B) and *p*-JNK (D) showed significant suppression in concentration-dependent manner whereas *p*-ERK showed a significant suppression with all tested concentration of CAME (C). The data presented are the means ± SD of three independent experiments. *P < *0.05*, **P < *0.01* vs. PMA/A23187 alone.

MAPKs signaling pathways have been reported to be involved in the degranulation of activated mast cells [20,29]. The expression level of MAPKs was measured in the presence of PMA/A23187 to examine the effect of MAPKs on mast cell activation and then the effect of CAME was measured on PMA/A23187-induced MAPKs expression in RBL-2H3 cells. PMA/A23187 challenge resulted in the increased phosphorylation of p38, ERK, and JNK in RBL-2H3 cells (Fig. 4). CAME treatment significantly attenuated the PMA/A23187-induced phosphorylation of all three MAPKs (Fig. 4). Quantitative analyses of MAPK immunoblots showed a significant inhibition in a concentration-dependent (Fig. 4B, C & 4D).

### 3.5 CAME suppressed PMA/A23187-induced nuclear translocation of NF-κB

**Fig. 5:**
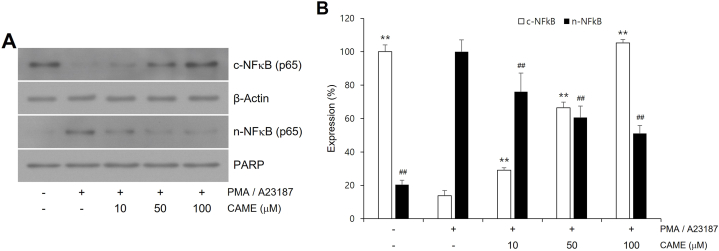
Effects of CAME on PMA/A23187-induced nuclear NFκB and cytosolic NFκB in RBL-2H3 cells. (A) Representative immunoblots. (B) Quantitative analyses of immunoblots of nuclear NFκB and cytosolic NFκB. Cells were pretreated with CAME (10, 50, and 100 μM) for 1 h and then stimulated with PMA/A23187 for 30min. CAME significantly suppressed nuclear translocation of NFκB in a concentration-dependent manner. The data presented are the means ± SD of three independent experiments. **P < *0.01* vs. PMA/A23187 alone in cytosolic fraction; #P < 0.01 vs PMA/A23187 alone in nuclear fraction.

NF-κB has been known to be a major transcription factor of pro-inflammatory genes in mast cells [27]. In the present study, the effect of CAME on PMA/A23187-induced nuclear translocation of NF-κB was examined in RBL-2H3 cells. PMA/A23187 treatment exhibited significantly increased nuclear translocation of NF-κB in RBL-2H3 cells (Fig. 5). Representative immunoblot showed almost complete nuclear translocation of NF-κB upon PMA/A23187 treatment. However, CAME treatment significantly suppressed PMA/A23187-induced nuclear translocation of NF-κB in a concentration-dependent manner (Fig. 5). With CAME treatment, the level of nuclear NF-κB was reversely correlated with the level of cytosolic NF-κB (Fig. 5).

## 4 Discussion

The present study clearly demonstrate that CAME inhibited PMA/A23187-induced allergic responses in RBL-2H3 mast cells. CAME significantly suppressed PMA/A23187-induced secretion of TNF-α, hexosaminidase, and histamine in PMA/A23187-challenged RBL-2H3 cells. CAME significantly attenuated PMA/A23187-induced COX-2 expression, MAPKs phosphorylation, and nuclear translocation of NF-κB in RBL-2H3 cells.

Allergic disorders such as asthma, atopic dermatitis, rhinitis and anaphylaxis, are hypersensitive reaction of the immune system and characterized by the infiltration and the accumulation of lymphocytes, basophils, eosinophils and mast cells at the site of inflammation [1]. Among these cells, aberrantly activated mast cells play a detrimental role in the propagation of allergic response [3,4]. Once activated, mast cell release chemotactic and inflammatory cytokines such as TNF-α and interleukins, and immunoregulatory mediators such as histamine, proteases, and prostaglandins [30]. These inflammatory cytokines and mediators consequently initiate the immediate hypersensitivity reaction such as leukocyte recruitment, vasodilation, increased vascular permeability and bronchoconstriction causing symptoms such as asthmatic attack and anaphylactic shock [31,32]. Especially, TNF-α has been known to be involved in leukocyte recruitment [33]. Accordingly, the release of TNF-α was examined in PMA/A23187-challenged RBL-2H3 cells in the present study. CAME significantly inhibited PMA/A23187-induced TNF-α release in a concentration-dependent manner (Fig. 1). Given that degranulation of mast cell has been reported to be quantitatively determined by measuring the level of released β-hexosaminidase [34] and histamine is the most well characterized and most potent vasoactive mediator in activated mast cells [21], secretion of β-hexosaminidase and histamine was quantitatively measured in the present study. PMA/A23187-challenged RBL-2H3 cells showed the increased release of β-hexosaminidase and histamine and CAME significantly suppressed PMA/A23187-induced secretion of β-hexosaminidase and histamine in RBL-2H3 cells in a concentration-dependent manner (Fig. 2). Activated mast cells also exhibit the upregulation of pro-inflammatory gene expression of COX-2, which results in the production of prostaglandin E2 that causes increased vascular permeability contributing to the aggravation of inflammation [35]. PMA/A23187-challenged showed increased expression of COX-2 and CAME significantly suppressed PMA/A23187-induced COX-2 expression in PMA/A23187-challenged RBL-2H3 cells (Fig. 3).

MAPKs participate in the immune response of mast cells [13,36]. The production of pro-inflammatory cytokines and mediators during the mast cell activation has been demonstrated to be associated with MAPKs signaling pathway [6,20,29]. Treatment of PMA and calcium ionophore to mast cells results in the activation of MAPK signaling pathways including p38, ERK, and JNK and subsequent expression of pro-inflammatory mediators including cytokines [37,38]. Coumarin derivative has been reported to suppress PMA/A21378-induced allergic inflammation by inhibiting ERK signaling pathway in RBL-2H3 cells [39]. CAME has been reported to inhibit pathologic allergic response by inhibiting JNK activation in HMC-1 human mast cells [21]. In addition, bis-dimethoxycoumarin has been demonstrated to inhibit JNK, ERK and p38 MAPKs and attenuates allergic response in PMA/A21378-induced HMC-1 human mast cells [6]. These reports indicate that MAPKs signaling pathway is clearly involved in the progression of allergic response in abnormally activated mast cells. In the present study, PMA/A21378 treatment results in the phosphorylation of all three MAPKs and CAME treatment exhibited the significant inhibition of MAPKs phosphorylation in a concentration-dependent manner (Fig. 4).

NF-κB is the major transcription factor for the expression of a variety of pro-inflammatory mediators such as iNOS, IL-1β, and TNF-α and the expression of adhesion molecules [40,41]. The aberrant activation of NF-κB has been widely demonstrated to be associated with various pathologic conditions including autoimmune diseases [42]. NF-κB is retained in the cytosol due to the sequestering interaction with IκB in the absence of stimuli, but pro-inflammatory signals such as LPS result in the degradation of IκB and freed NF-κB translocates to the nucleus leading to the expression of pro-inflammatory genes [43]. Inflammatory stimuli such as LPS cause nuclear translocation of NF-κB in various immune cells [44,45]. In the present study, nuclear translocation of NF-κB was observed with PMA/A21387 treatment in RBL-2H3 cells and CAME treatment significantly attenuated PMA/A21387-induced nuclear translocation of NF-κB (Fig. 5). With CAME treatment, NF-κB was maintained in the cytosol presumably due to the inhibition of IκB degradation in a concentration-dependent manner. MAPKs has been reported to mediate nuclear translocation of NF-κB through controlling the degradation of IκB [46].

Caffeic acids and their derivatives have been reported to possess a wide range of biological properties such as antitumor, anti-inflammatory, immunosuppressive, antimicrobial and neuroprotective properties [[47], [48], [49]]. THC, a caffeic acid derivative, exhibited anti-inflammatory action through the inhibition of NF-κB activation in LPS-challenged BV2 microglial cells [27], and through the activation of Nrf2/HO-1 signaling pathway in RAW264.7 macrophage cells [50]. THC suppressed LPS-induced macrophage infiltration to kidney and exhibited improved survival of mice in LPS-induced endotoxemia model [50,51]. Furthermore, THC exerted anti-inflammatory effects on atopic dermatitis model in human keratinocyte cell line, HaCat cells [52]. CAPE showed anti-inflammatory effect by inhibiting JNK phosphorylation and NF-κB signaling in HMC-1 human mast cells [21]. CAME exhibited anti-inflammatory effect through the inhibition of prostaglandin E2, nitric oxide, and TNF-α production in LPS-challenged RAW264.7 cells [22]. It would be informative to examine anti-allergic effects of caffeic acid derivatives such as CAME, CAPE, and other derivatives together to compare the relative anti-allergic effect of these derivatives.

In conclusion, in addition to our previous reports that THC, a caffeic acid derivative, exerts anti-inflammatory response in microglial, macrophage, and keratinocyte cells [[50], [51], [52]], and endotoxemia animal model [50,51], the present data clearly demonstrate that CAME significantly suppresses PMA/A23187-induced mast cell activation through the inhibition of MAPKs and NF-κB signaling pathways in BRL-2H3 cells, suggesting that CAME could be a valuable therapeutic agent in the control of various allergy-related disorders.

## Author contribution statement

Jinyoung Park, Hee Jae Lee, Wanjoo Chun: Conceived and designed the experiments; Performed the experiments; Analyzed and interpreted the data; Contributed reagents, materials, analysis tools or data; Wrote the paper. Eun-Taek Han, Jin-Hee Han: Performed the experiments; Contributed reagents, materials, analysis tools or data. Won Sun Park, Yong-Soo Kwon: Analyzed and interpreted the data; Contributed reagents, materials, analysis tools or data.

## 5 Data availability statement

Data will be made available on request.

## Funding statement

This work was supported by the 10.13039/501100003725National Research Foundation of Korea (NRF), South Korea grant funded by the Korea government [2021-R1A4A1031574].

## Declaration of competing interest

The authors declare that they have no known competing financial interests or personal relationships that could have appeared to influence the work reported in this paper.
